# Supplementary material for: Assessing knowledge about hypertension and identifying predictors of inadequate knowledge in Saudi Arabia: A cross-sectional study
Source: PLoS One. 2024 Mar 18;19(3):e0299745. doi: 10.1371/journal.pone.0299745 (PMC10947669; doi:10.1371/journal.pone.0299745)
Supplement: S1 Table — STROBE Statement—checklist of items that should be included in reports of observational studies. (DOCX) [file pone.0299745.s002.docx]

S1 Table: STROBE Statement—checklist of items that should be included in reports of observational studies.

|  | **Item No.** | **Recommendation** | **Page No.** | **Relevant text from manuscript** |
| --- | --- | --- | --- | --- |
| **Title and abstract** | 1 | (*a*) Indicate the study’s design with a commonly used term in the title or the abstract | 2 | A cross-sectional study was conducted in the Jazan region between February and April 2023. |
|  |  | (*b*) Provide in the abstract an informative and balanced summary of what was done and what was found | 2 | Abstract |
| **Introduction** | | | |  |
| Background/rationale | 2 | Explain the scientific background and rationale for the investigation being reported | 3-5 | Given that knowledge of hypertension management plays a significant role in prevention and control, research that investigating this knowledge in hypertensive patients is vital for public health… |
| Objectives | 3 | State specific objectives, including any prespecified hypotheses | 5 | This study aimed to assess overall and specific knowledge areas of hypertension and to identify the predictors of inadequate levels of knowledge of hypertensive patients in the Jazan region of Saudi Arabia. |
| **Methods** | | | |  |
| Study design | 4 | Present key elements of study design early in the paper | 5 | A cross-sectional study was conducted using an online, self-administered questionnaire disseminated to hypertensive patients in the Jazan region from February to April 2023. |
| Setting | 5 | Describe the setting, locations, and relevant dates, including periods of recruitment, exposure, follow-up, and data collection | 5-6 | Study design and settings subsection, study participants and eligibility criteria subsection |
| Participants | 6 | (*a*) *Cohort study*—Give the eligibility criteria, and the sources and methods of selection of participants. Describe methods of follow-up  *Case-control study*—Give the eligibility criteria, and the sources and methods of case ascertainment and control selection. Give the rationale for the choice of cases and controls  *Cross-sectional study*—Give the eligibility criteria, and the sources and methods of selection of participants | 6 | Study participants and eligibility criteria subsection |
|  |  | (*b*) *Cohort study*—For matched studies, give matching criteria and number of exposed and unexposed  *Case-control study*—For matched studies, give matching criteria and the number of controls per case | NA |  |
| Variables | 7 | Clearly define all outcomes, exposures, predictors, potential confounders, and effect modifiers. Give diagnostic criteria, if applicable | 8-9 | Statistical analysis subsection |
| Data sources/ measurement | 8* | For each variable of interest, give sources of data and details of methods of assessment (measurement). Describe comparability of assessment methods if there is more than one group | 7 | Data collection instrument subsection |
| Bias | 9 | Describe any efforts to address potential sources of bias | No |  |
| Study size | 10 | Explain how the study size was arrived at | 6 | Study participants and eligibility criteria subsection |

Continued on next page

| Quantitative variables | 11 | Explain how quantitative variables were handled in the analyses. If applicable, describe which groupings were chosen and why | 8-9 | Statistical analysis subsection |
| --- | --- | --- | --- | --- |
| Statistical methods | 12 | (*a*) Describe all statistical methods, including those used to control for confounding | 8-9 | Statistical analysis subsection |
|  |  | (*b*) Describe any methods used to examine subgroups and interactions | No |  |
|  |  | (*c*) Explain how missing data were addressed | NA | No missing data |
|  |  | (*d*) *Cohort study*—If applicable, explain how loss to follow-up was addressed  *Case-control study*—If applicable, explain how matching of cases and controls was addressed  *Cross-sectional study*—If applicable, describe analytical methods taking account of sampling strategy | 6 | Study participants and eligibility criteria subsection |
|  |  | (*e*) Describe any sensitivity analyses | No |  |
| **Results** | | | | |
| Participants | 13* | (a) Report numbers of individuals at each stage of study—eg numbers potentially eligible, examined for eligibility, confirmed eligible, included in the study, completing follow-up, and analysed | 9 | Characteristic of hypertensive participants subsection |
|  |  | (b) Give reasons for non-participation at each stage | 9 | Characteristic of hypertensive participants subsection |
|  |  | (c) Consider use of a flow diagram | No |  |
| Descriptive data | 14* | (a) Give characteristics of study participants (eg demographic, clinical, social) and information on exposures and potential confounders | 9-10 | [Table 1] |
|  |  | (b) Indicate number of participants with missing data for each variable of interest | NA | No missing data |
|  |  | (c) *Cohort study*—Summarise follow-up time (eg, average and total amount) | NA |  |
| Outcome data | 15* | *Cohort study*—Report numbers of outcome events or summary measures over time | NA |  |
|  |  | *Case-control study—*Report numbers in each exposure category, or summary measures of exposure | NA |  |
|  |  | *Cross-sectional study—*Report numbers of outcome events or summary measures | 9-14 | [Table 1-4] |
| Main results | 16 | (*a*) Give unadjusted estimates and, if applicable, confounder-adjusted estimates and their precision (eg, 95% confidence interval). Make clear which confounders were adjusted for and why they were included | 14 | [Table 4] |
|  |  | (*b*) Report category boundaries when continuous variables were categorized | 9-14 | [Table 1-4]: Age group |
|  |  | (*c*) If relevant, consider translating estimates of relative risk into absolute risk for a meaningful time period | NA |  |

| Other analyses | 17 | Report other analyses done—eg analyses of subgroups and interactions, and sensitivity analyses | S3-5 Tables | Supporting information section |
| --- | --- | --- | --- | --- |
| **Discussion** | | | | |
| Key results | 18 | Summarise key results with reference to study objectives | 14-16 | We found that the overall mean score of knowledge levels of participants was 17.60 (±5.09), which represents (67.7%) of the maximum score. The majority of participants (59.3%); (95% CI: 53.0%-66.0%) were classified as having an inadequate knowledge about the disease and its management… |
| Limitations | 19 | Discuss limitations of the study, taking into account sources of potential bias or imprecision. Discuss both direction and magnitude of any potential bias | 18 | This study has several limitations… |
| Interpretation | 20 | Give a cautious overall interpretation of results considering objectives, limitations, multiplicity of analyses, results from similar studies, and other relevant evidence | 18 | Conclusion beside discussion sections |
| Generalisability | 21 | Discuss the generalisability (external validity) of the study results | 18 | Second, using non-probability sampling strategy affected the study’s external validity, which could limit its generalizability. |
| **Other information** | |  | | |
| Funding | 22 | Give the source of funding and the role of the funders for the present study and, if applicable, for the original study on which the present article is based | Yes | Financial support statement |

*Give information separately for cases and controls in case-control studies and, if applicable, for exposed and unexposed groups in cohort and cross-sectional studies.

**Note:** An Explanation and Elaboration article discusses each checklist item and gives methodological background and published examples of transparent reporting. The STROBE checklist is best used in conjunction with this article (freely available on the Web sites of PLoS Medicine at http://www.plosmedicine.org/, Annals of Internal Medicine at http://www.annals.org/, and Epidemiology at http://www.epidem.com/). Information on the STROBE Initiative is available at www.strobe-statement.org.
