# Supplementary material for: Assessing knowledge about hypertension and identifying predictors of inadequate knowledge in Saudi Arabia: A cross-sectional study
Source: PLoS One. 2024 Mar 18;19(3):e0299745. doi: 10.1371/journal.pone.0299745 (PMC10947669; doi:10.1371/journal.pone.0299745)
Supplement: S2 Table — Modifications applied to the HK-LS. (DOCX) [file pone.0299745.s003.docx]

| **Subdimension** | **Number of added items** | **Aspects of assessment of the added items** | **Total number of items** | **α** | **Overall HK-LS (α)** |
| --- | --- | --- | --- | --- | --- |
| Disease definition | 1 | Isolated systolic hypertension | 3 | 0.782 | 0.932 |
| Medical treatment | 0 | Not applicable | 4 | 0.450 |  |
| Drug compliance | 0 | Not applicable | 4 | 0.854 |  |
| Lifestyle | 3 | Risk of increased salt consumption, obesity and the recommended time for exercise per week | 8 | 0.862 |  |
| Diet | 0 | Not applicable | 2 | 0.845 |  |
| Complications | 0 | Not applicable | 5 | 0.757 |  |
| α: Cronbach's alpha. | | | | | |

S3 Table: Modifications applied to the HK-LS.
