# Supplementary material for: Assessing knowledge about hypertension and identifying predictors of inadequate knowledge in Saudi Arabia: A cross-sectional study
Source: PLoS One. 2024 Mar 18;19(3):e0299745. doi: 10.1371/journal.pone.0299745 (PMC10947669; doi:10.1371/journal.pone.0299745)
Supplement: S3 Table — Tests of normality of dependent variables. (DOCX) [file pone.0299745.s004.docx]

S4 Table: Tests of normality of the dependent variables.

| **Dependent variables** | **Kolmogorov-Smirnov** | | |
| --- | --- | --- | --- |
|  | **Statistic** | **df** | ***P*-value** |
| Overall HK-LS | 0.088 | 253 | <0.001 |
| Disease definition subdimension | 0.290 | 253 | <0.001 |
| Medical treatment subdimension | 0.210 | 253 | <0.001 |
| Drug compliance subdimension | 0.279 | 253 | <0.001 |
| Lifestyle subdimension | 0.251 | 253 | <0.001 |
| Diet subdimension | 0.240 | 253 | <0.001 |
| Complications subdimension | 0.241 | 253 | <0.001 |
