# Supplementary material for: Assessing knowledge about hypertension and identifying predictors of inadequate knowledge in Saudi Arabia: A cross-sectional study
Source: PLoS One. 2024 Mar 18;19(3):e0299745. doi: 10.1371/journal.pone.0299745 (PMC10947669; doi:10.1371/journal.pone.0299745)
Supplement: S4 Table — Mean ranks of nonparametric tests. (DOCX) [file pone.0299745.s005.docx]

S5 Table: Mean ranks of nonparametric tests.

| **Variables** | **HK-LS Subdimensions (Mean Rank)** | | | | | | |
| --- | --- | --- | --- | --- | --- | --- | --- |
|  | **Disease Definition** | **Medical Treatment** | **Drug Compliance** | **Lifestyle** | **Diet** | **Complications** | **Overall Scale** |
| **Sex** | | | | | | | |
| Male | 128.52 | 142.93 | 122.71 | 136.84 | 123.82 | 131.57 | 133.81 |
| Female | 126.02 | 116.76 | 129.76 | 120.67 | 129.05 | 124.06 | 122.62 |
| *P*-value^a^ | 0.778 | 0.004 | 0.435 | 0.075 | 0.556 | 0.403 | 0.234 |
| **Age group** | | | | | | | |
| <35 years | 119.73 | 105.26 | 109.28 | 108.86 | 109.89 | 123.11 | 104.32 |
| 35-44 years | 127.47 | 121.27 | 122.35 | 125.23 | 124.11 | 129.49 | 120.63 |
| 45-54 years | 118.93 | 132.65 | 129.98 | 132.39 | 144.36 | 130.08 | 134.19 |
| 55-64 years | 139.91 | 136.66 | 146.01 | 138.37 | 128.37 | 122.61 | 142.22 |
| ≥65 years | 128.98 | 156.73 | 135.06 | 138.45 | 139.39 | 131.42 | 148.73 |
| *P*-value^b^ | 0.550 | 0.012 | 0.084 | 0.185 | 0.119 | 0.954 | 0.022 |
| **Educational level** | | | | | | | |
| No formal education | 104.37 | 100.10 | 91.91 | 109.09 | 107.44 | 102.91 | 89.96 |
| Primary | 97.82 | 96.35 | 118.09 | 116.71 | 140.00 | 132.41 | 110.12 |
| Intermediate | 119.58 | 134.63 | 124.57 | 122.48 | 126.54 | 123.54 | 122.13 |
| Secondary | 122.41 | 132.84 | 127.14 | 128.36 | 116.94 | 132.88 | 125.80 |
| Higher education | 146.59 | 139.30 | 145.64 | 138.44 | 137.19 | 137.03 | 149.24 |
| *P*-value^b^ | 0.001 | 0.005 | <0.001 | 0.158 | 0.103 | 0.07 | <0.001 |
| **Residency area** | | | | | | | |
| Urban | 124.68 | 122.24 | 122.45 | 127.26 | 125.18 | 131.90 | 125.17 |
| Rural | 129.10 | 131.30 | 131.10 | 126.76 | 128.64 | 122.58 | 128.65 |
| *P*-value^a^ | 0.609 | 0.304 | 0.326 | 0.955 | 0.690 | 0.288 | 0.705 |
| **History of complications** | | | | | | | |
| Negative | 122.89 | 117.85 | 122.43 | 119.80 | 126.23 | 114.36 | 116.61 |
| Positive | 130.82 | 135.52 | 131.26 | 133.71 | 127.72 | 138.77 | 136.68 |
| *P*-value^a^ | 0.359 | 0.045 | 0.316 | 0.117 | 0.864 | 0.005 | 0.029 |
| **Duration since diagnosis** | | | | | | | |
| Since less than 2 years | 122.31 | 115.19 | 116.03 | 114.08 | 127.57 | 134.04 | 117.76 |
| Since 2-5 years | 135.02 | 119.61 | 135.38 | 130.46 | 124.00 | 120.10 | 128.10 |
| Since 6-10 years | 127.73 | 135.95 | 131.91 | 137.27 | 130.30 | 112.70 | 131.60 |
| Since more than 10 years | 123.17 | 145.88 | 128.03 | 130.80 | 125.49 | 144.33 | 135.54 |
| *P*-value^b^ | 0.710 | 0.062 | 0.359 | 0.226 | 0.961 | 0.074 | 0.535 |
| ^a^Mann-Whitney U test, ^b^Kruskal-Wallis H test. | | | | | | | |
