# Supplementary material for: Assessing knowledge about hypertension and identifying predictors of inadequate knowledge in Saudi Arabia: A cross-sectional study
Source: PLoS One. 2024 Mar 18;19(3):e0299745. doi: 10.1371/journal.pone.0299745 (PMC10947669; doi:10.1371/journal.pone.0299745)
Supplement: Attachment — Submitted filename: Reviewer comments.docx [file pone.0299745.s006.docx]

Reviewer comments

Overall, the paper titled ‘Assessing knowledge about hypertension and identifying predictors of inadequate knowledge in the Jazan region is a well written scholarly document. Although it attempted to address this important topic there are some critical issues that warrant further attention. These would improve the research's quality and robustness, making it more academic journal-worthy. Points for improvement:

1. Title and Abstract: Text clarity, conciseness and structure can be improved.

2. Literature assessment: A more thorough assessment would strengthen the study's scientific foundation. This would help understand the present state of knowledge and the research gaps the project attempts to address.

3. Methods: More detailed information about the tool for data would be helpful.

4.Discussion: Attention and conciseness can be improved by emphasizing the most important facts and recommendations.
